# Canonical Wnt induction by OTULIN prevents keratinocyte death and skin inflammation

**DOI:** 10.1126/sciadv.adz0181

**Published:** 2026-10-09

**Authors:** Kim Lecomte, Pieter Hertens, Virginie Wylleman, Annagiada Toniolo, Febe Roelandt, Fleur Boone, Maarten Ciers, Lien Verboom, Katrien Staes, Geert van Loo, Esther Hoste

**Affiliations:** ^1^ VIB Center for Inflammation Research, 9052 Ghent, Belgium.; ^2^ Department of Biomedical Molecular Biology, Ghent University, 9052 Ghent, Belgium.; ^3^ Cancer Research Institute Ghent (CRIG), Ghent, Belgium.

## Abstract

Loss-of-function mutations in the human *OTULIN* gene, encoding a deubiquitinase with exclusive specificity for linear ubiquitin chains, cause a severe multiorgan autoinflammatory condition involving the skin. Mice lacking OTULIN selectively in keratinocytes develop inflamed skin lesions that progress into squamous tumors, a phenotype driven by excessive tumor necrosis factor (TNF)–induced cell death. Previous studies suggest a role for OTULIN in mediating Wnt signaling during development, but the physiological relevance of this association is unknown. Here, we show that OTULIN promotes Wnt signaling in keratinocytes by regulating the linear ubiquitination status of β-catenin. Stabilization of β-catenin in OTULIN-deficient keratinocytes prevents progressive skin inflammation in prophylactic and therapeutic settings by blocking keratinocyte death. We demonstrate that OTULIN prevents proteasomal degradation of β-catenin in cultured keratinocytes. Reduced Wnt signaling in OTULIN-deficient keratinocytes leads to degradation of TCF3/4, an essential survival factor for keratinocytes. Collectively, our data identify OTULIN’s linear deubiquitination activity as a key regulator of epithelial cell viability, not only by preventing cell death downstream of TNF, but also by promoting canonical Wnt signaling.

## INTRODUCTION

Keratinocytes (KCs), the epithelial cells of the skin, constitute a crucial structural and immunological barrier protecting the organism from dehydration and external hazards (*1*). The survival of KCs under environmental stress critically depends on their precise regulation of inflammatory signaling cascades, which are largely mediated through specific and fast posttranslational modifications of signaling proteins, ultimately triggering downstream transcriptional responses (*2*). Ubiquitination is a posttranslational modification that plays a key role in orchestrating cellular responses in inflammation. Linear (M1-linked) ubiquitin (Ubq) chains are head-to-tail Ubq moieties important for assembling protein complexes downstream of tumor necrosis factor (TNF), interleukin-1β (IL-1β) and Toll-like receptors (*3*–*6*). M1-linked Ubq chains are added to substrates by the linear ubiquitin chain assembly complex (LUBAC), consisting of heme-oxidized IRP2 ubiquitin ligase-1 (HOIL-1), HOIL interacting protein-1 (HOIP), and shank-associated RH domain-interacting protein (SHARPIN) (*7*). These M1 Ubq chains can be removed from substrates by the deubiquitinases (DUBs) OTU DUB with linear linkage specificity (OTULIN, encoded by the gene *Fam105b*, also known as *gumby*) and cylindromatosis (CYLD). OTULIN is the only DUB with exclusive specificity for M1-linked Ubq chains, whereas CYLD is also capable of removing Lys63 (K63)-linked Ubq chains (*8*–*10*). OTULIN prevents LUBAC autoubiquitination and degradation, stabilizing LUBAC in specific cell types, including KCs, thereby regulating NF-κB signaling and preventing cell death (*11*–*15*).

Patients harboring loss-of-function mutations in *OTULIN* develop a severe, often lethal, disease with skin involvement, termed ORAS (OTULIN-related autoinflammatory syndrome), and can be successfully treated with TNF-blocking therapies (*11*, *16*). In mice, genetic deletion of *Otulin* or expression of a catalytically inactive OTULIN mutant results in embryonic lethality due to excessive cell death (*11*, *14*, *17*). We and others have previously shown that mice lacking OTULIN selectively in KCs develop severe skin inflammation driven by TNFR1 signaling (*12*, *13*). Rivkin *et al.* identified the *Otulin/gumby* gene as causative for the recessive, embryonically lethal gumby mouse mutant and suggested a critical role for the gumby-LUBAC axis in regulating canonical Wnt signaling (*17*). However, direct in vivo evidence supporting such a role is currently lacking. Wnt signaling pathways regulate a diverse range of cellular processes, including cell fate determination, proliferation, and migration (*18*). Wnt is crucial for timely adaption of embryonic and adult stem cell behavior and induces hair follicle (HF) stem cell proliferation in the skin, while also controlling the commitment and differentiation of other KCs (*19*, *20*). Canonical Wnt signaling converges on cytoplasmic β-catenin accumulation. In absence of Wnt ligands, β-catenin is bound by a destruction complex comprising glycogen synthase-kinase-3β (GSK3β), axis inhibition 2 (AXIN2), adenomatous polyposis coli (APC), and casein kinase 1α (CK1α), resulting in β-catenin phosphorylation. Phosphorylated β-catenin is subsequently recognized by β-transducin repeat containing E3 Ubq protein ligase (β-TrCP), which catalyzes the addition of K48-linked Ubq chains, thereby targeting β-catenin for proteasomal degradation (*21*). Upon binding of Wnt ligands to Frizzled (Fzd) receptors and low-density lipoprotein receptor-related protein 5/6 (LRP5/6), the adaptor protein Dishevelled2 (DVL2) is recruited to the membrane and binds AXIN2, thereby disrupting the destruction complex. This disruption allows β-catenin to accumulate in the cytosol and translocate to the nucleus, where it activates transcription of Wnt target genes by forming a complex with members of the T cell factor (TCF)/lymphoid enhancer–binding factor (LEF) family of transcription factors (*22*). The E3 Ubq ligases RNF43 and ZNRF3 function as Wnt repressors by catalyzing K48-linked ubiquitination, triggering the turnover or endocytosis of Fzd receptors (*23*, *24*), whereas β-catenin degradation is predominantly mediated by the SCF E3 ligase complex containing β-TrCP (*21*, *25*). Overexpression studies in cancer cell lines suggested that linear ubiquitination of β-catenin promotes its K48-linked ubiquitination and degradation in response to DNA damage, a process counteracted by OTULIN (*26*).

In this study, we elucidate the in vivo importance of OTULIN in regulating canonical Wnt signaling in KCs. We demonstrate that stabilization of β-catenin and expression of the Wnt target genes *Tcf3* and *Tcf4* depends on OTULIN’s DUB activity, thereby protecting KCs from TNF-induced cell death and severe skin inflammation in mice. Notably, stabilizing β-catenin in OTULIN-deficient KCs prevents or ameliorates skin inflammation in prophylactic and therapeutic settings, respectively. These findings provide important insights into how morphogen signaling intersects with cell death pathways to safeguard epithelial cell survival.

## RESULTS

### Wnt signaling is down-regulated in OTULIN-deficient KCs

OTULIN was initially identified as a modulator of canonical Wnt signaling regulating embryonic angiogenesis (*17*). To determine whether OTULIN and linear (de)ubiquitination modulate Wnt responses in adult tissues, we analyzed Wnt signaling in the skin of mice with OTULIN-deficient KCs (∆^Ker^OTULIN mice). We previously demonstrated that ∆^Ker^OTULIN mice develop inflamed skin lesions that progress into well-differentiated squamous carcinomas (*12*, *13*). Gene ontology (GO) enrichment analysis of our published single-cell RNA sequencing (scRNA-seq) dataset comparing total skin of wild-type (WT; Cre-negative OTULIN^FL/FL^) and ∆^Ker^OTULIN mice (*12*) revealed Wnt signaling as one of the top down-regulated GO terms in OTULIN-deficient KCs (Fig. 1A and fig. S1A). When probing expression levels of specific Wnt mediators in our scRNA-seq dataset, we indeed observed marked down-regulation in OTULIN-deficient KCs relative to control KCs (Fig. 1B). Quantitative polymerase chain reaction (qPCR) analysis of epidermal tail lysates confirmed the down-regulation of Wnt target genes in ∆^Ker^OTULIN epidermis (Fig. 1C and Table 1). In agreement, expression levels of several members of the TCF/LEF family, key downstream mediators of canonical Wnt signaling, were also significantly reduced in ∆^Ker^OTULIN epidermis (Fig. 1D). Immunoblotting for nonphosphorylated β-catenin, indicative of stabilized β-catenin and active Wnt signaling, confirmed reduced Wnt activation in epidermal tail lysates from ∆^Ker^OTULIN relative to control mice (Fig. 1E). Wnt activation in KCs from adult skin is most prominent during the growth phase of HFs, also referred to as anagen, culminating in nuclear expression of LEF1 (*20*, *27*). Immunofluorescence staining of anagen skin indicated nuclear LEF1 in WT HFs, whereas no nuclear LEF1 was observed in anagen-like HFs in lesional ∆^Ker^OTULIN skin (Fig. 1F and Table 2). When probing HF cell populations (outer and inner layer, bulge and upper HF) of our scRNA-seq data for Wnt target gene expression, the down-regulation of Wnt target gene expression in OTULIN-deficient KCs was even more pronounced than in the total KC population (Fig. 1G). In addition to OTULIN, SHARPIN has also been implicated in stabilizing β-catenin (*28*). Therefore, we quantified expression levels of Wnt mediators and target genes in SHARPIN^cpdm/cpdm^ epidermis and control epidermis. Down-regulation of canonical Wnt signaling was observed in SHARPIN^cpdm/cpdm^ epidermis, supporting a role for linear ubiquitination in promoting Wnt signaling in adult KCs (fig. S1B). β-catenin also performs Wnt-independent roles at the plasma membrane (*29*). We assessed the levels of cytosolic β-catenin in OTULIN-deficient and control epidermis and observed higher levels in control epidermis, indicating that OTULIN promotes canonical Wnt signaling in the skin (fig. S1C). Together, these data demonstrate that OTULIN promotes canonical Wnt signaling in adult KCs.

**Fig. 1.**
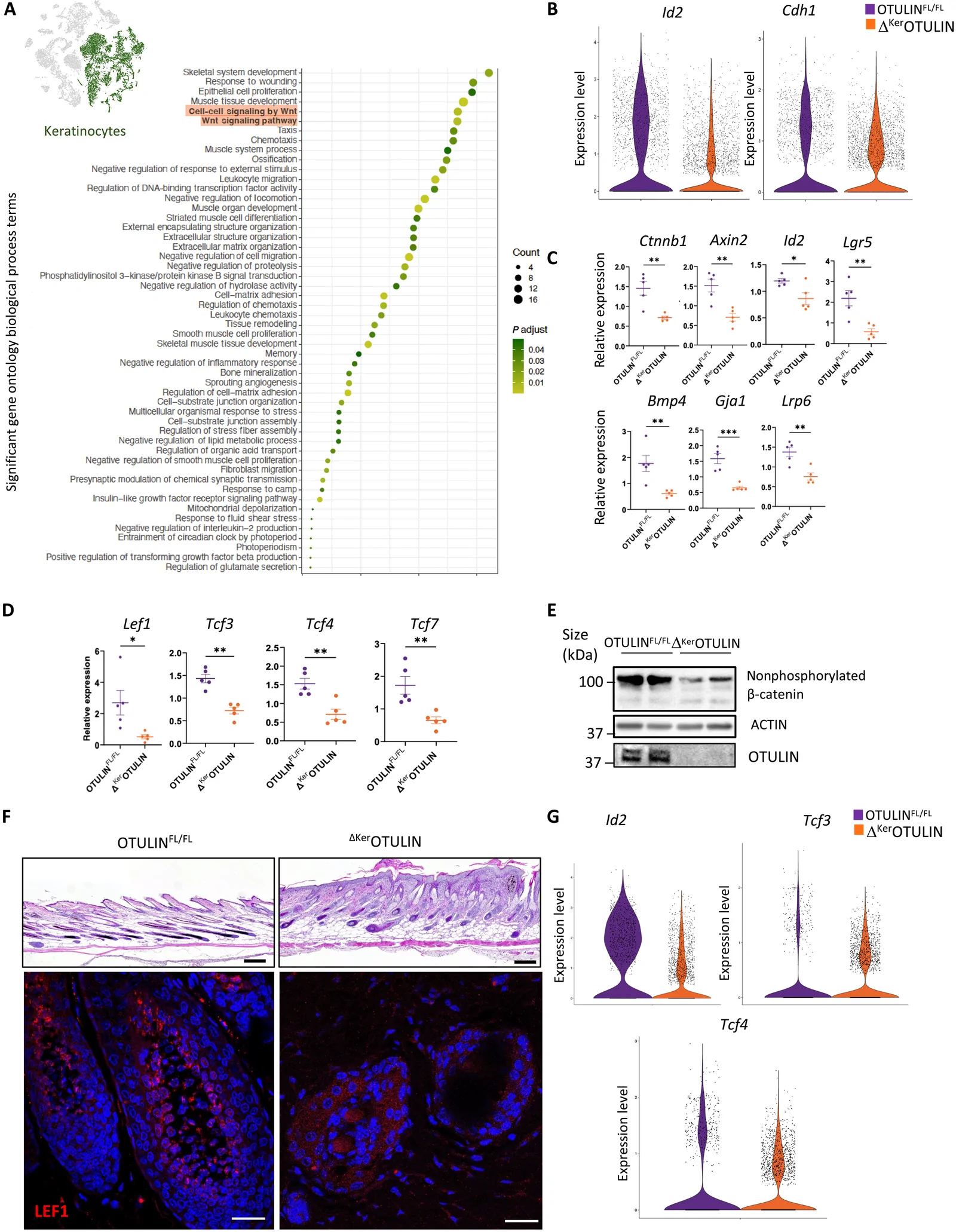
Wnt signaling is down-regulated in OTULIN-deficient KCs. (**A**) Left: UMAP clustering of scRNA-seq data obtained from cells isolated from ∆^Ker^OTULIN and control skin (*12*), highlighting the KC cluster. Right: GO enrichment analysis of genes significantly down-regulated in OTULIN-deficient KCs relative to control KCs. (**B**) Violin plots showing distribution of expression of the indicated Wnt target genes in scRNA-seq data of KCs from OTULIN^FL/FL^ and ∆^Ker^OTULIN skin. (**C**) qPCR analysis for the indicated Wnt target genes on epidermal tail lysates of OTULIN^FL/FL^ and Δ^Ker^OTULIN mice (aged 8 to 11 weeks). Data are means ±SEM, ^∗^*P* < 0.05; ^∗∗^*P* < 0.01; ^∗∗∗^*P* < 0.001. (**D**) qPCR analysis for the indicated Wnt effector genes on epidermal tail lysates of OTULIN^FL/FL^ and Δ^Ker^OTULIN mice. Data are means ± SEM, ^∗^*P* < 0.05; ^∗∗^*P* < 0.01; ^∗∗∗^*P* < 0.001. Violin plots showing distribution of expression of the indicated genes in scRNA-seq data of HF KCs from OTULIN^FL/FL^ and ∆^Ker^OTULIN skin. (**E**) Immunoblotting for nonphosphorylated (active) β-catenin and OTULIN in epidermal tail lysates from OTULIN^FL/FL^ and ∆^Ker^OTULIN mice. α-Actin is shown as loading control. (**F**) Immunofluorescence images of LEF1 staining in OTULIN^FL/FL^ anagen skin and ∆^Ker^OTULIN lesional skin. Top panels are hematoxylin and eosin (H&E)–stained skin sections from the same mice. Scale bars, 100 μm (top); 50 μm (bottom). (**G**) Violin plots showing distribution of expression of the indicated genes in scRNA-seq data of HF KCs from OTULIN^FL/FL^ and ∆^Ker^OTULIN skin.

**Table 1.** List of mouse-specific qPCR primers.

Gene	Forward primer	Reverse primer
*Axin2*	CTCACCCATTTACCCAGGACC	GTTCCTGTCCCTCTGCTGAC
*Ctnnb*	CACAGCTCCTTCCCTGAGTG	GCCCTCATCTAGCGTCTCAG
*Bmp4*	CCTGCAGCGATCCAGTCTCT	ACTACGGAATGGCTCCATT
*Gja1*	CCAAGGAGTTCCACCACTTTG	CCATGTCTGGGCACCTCTCT
*Id2*	CTGGGCAAGACGATCATCCTT	CCCTTCTGAGCTTATGTCGAATG
*Lef1*	GAT CCC CTT CAA GGA CGA AG	GGC TTG TCT GAC CAC CTC AT
*Lgr5*	TCTTCACCTCCTACCTGGACCT	GGCGTAGTCTGCTATGTGGTGT
*Lrp6*	CTGAATGCTGACAACAGGACCTG	GACGTTCCGAAGGCTGTGGATA
*Tcf3*	CCATGCTAGGTGACGGCTCTTC	GCGAGCCATTAACCTCAGATCC
*Tcf4*	CCTCCAATCCTTCAACTCCTGTG	TCCAAACGGTCTTCGATTCGGC
*Tcf7*	ATCCTTGATGCTGGGATTCTG	CTTCTCTTGCCTTGGGTTCTG
*Hprt1*	AGTGTTGGATACAGGCCAGAC	CGTGATTCAAATCCCTGAAGT
*Matrin3*	TGG ACC AAG AGG AAA TCT GG	TGA ACA ACT CGG CTG GTT TC
*Eef1a1*	TCGCCTTGGACGTTCTTTT	GTGGACTTGCCGGAATCTAC

**Table 2.** List of antibodies.

Antibody	Concentration	Manufacture	Catalog number	Application
Cleaved caspase-3	1/500	Cell Signaling Technology	9661	Immunohistochemistry
				Immunofluorescence
Cleaved caspase-8	1/500	Cell Signaling Technology	9429	Immunofluorescence
Ki67	1/300	Invitrogen	14-5698-82	Immunohistochemistry
Tcf4	1/100	Santa Cruz Biotechnology	SC166699	Immunofluorescence
Lef1	1/200	Cell Signaling Technology	2230S	Immunofluorescence
K10	1/500	Invitrogen	MA5-42858	Immunofluorescence
K14	1/500	Santa Cruz Biotechnology	sc53253	Immunofluorescence
Tcf3	1/500	Cell Signaling	2883	Western blotting
β-Catenin	1/2000	BD Biosciences	610153	Western blotting
Non-P-β-catenin	1/1000	Merck	05-665	Western blotting
M1 Ubq	1/2000 or 1/10 000	Sigma-Aldrich	ZRB2114-4X25UL (clone 1E3)	Western blotting
OTULIN	1/1000	Cell Signaling Technology	14127	Western blotting
HOIP	1/1000	Gift of R. Damgaard		Western blotting
SHARPIN	1/1000	Proteintech	14626-1-AP	Western blotting
Caspase-3	1/1000	Cell Signaling Technology	9662	Western blotting
ACTIN-HRP	1/10000	Santa Cruz Biotechnology Biotechnology	sc-47778	Western blotting
Anti-rabbit HRP	1/2500	Amersham	NA931	Western blotting
Anti-mouse HRP	1/2500	Amersham	NA934	Western blotting
Anti-goat HRP	1/2500	Amersham	Sc-2354	Western blotting

### Stabilization of β-catenin prevents dermatitis and tumor formation in ∆^Ker^OTULIN mice

Canonical Wnt signaling is driven by cytosolic β-catenin accumulation and subsequent nuclear translocation, facilitating the interaction of β-catenin with TCF/LEF transcription factors to drive Wnt target gene expression (*22*). To assess whether inducing canonical Wnt signaling could compensate for the loss of OTULIN-driven Wnt activation, we stabilized β-catenin in KCs of ∆^Ker^OTULIN mice. Given the crucial role of Wnt signaling in embryogenesis, ∆^Ker^OTULIN mice were crossed to K14ΔNβ-catenin-ER mice (hereafter referred to as ΔNβcat mice), which express a transgene lacking the N-terminal degradome of β-catenin under the control of a KC-selective promoter following postnatal tamoxifen induction (*30*). Transgene induction in newborn ∆^Ker^OTULIN ΔNβcat pups at postnatal day 0.5 (P0.5) and P1.5 provided complete protection from dermatitis in the tail skin (Fig. 2A). Approximately 20% of these mice also exhibited full protection from dermatitis in the back skin, even at old age (fig. S2A). The remaining mice developed skin lesions, albeit significantly later than ∆^Ker^OTULIN mice (Fig. 2, A to C). Notably, all ∆^Ker^OTULIN ΔNβcat mice were protected from exophytic tumor formation (fig. S2B). Time course measurements of transepidermal water loss (TEWL) indicated that ∆^Ker^OTULIN mice exhibited more severe epidermal barrier perturbation compared to ∆^Ker^OTULIN ΔNβcat littermates (Fig. 2C). Clinical scoring of the mice further confirmed the significant improvement in skin pathology observed in ∆^Ker^OTULIN ΔNβcat relative to ∆^Ker^OTULIN mice (Fig. 2D). Additionally, protection from systemic autoinflammation in ∆^Ker^OTULIN ΔNβcat mice was evidenced by reduced levels of circulating IL-6 relative to ∆^Ker^OTULIN mice (Fig. 2E).

**Fig. 2.**
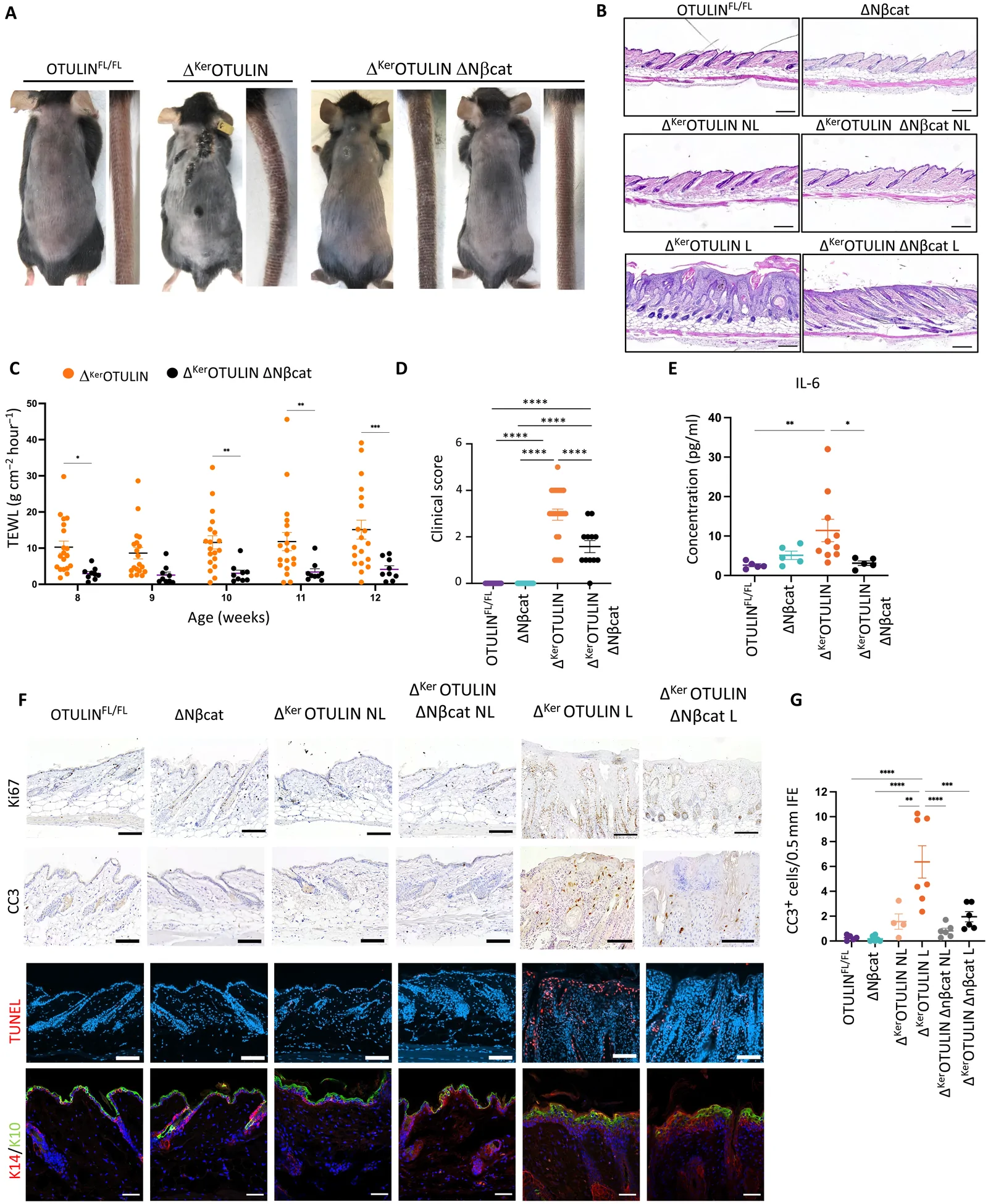
Stabilizing β-catenin in KCs protects Δ^Ker^OTULIN skin from dermatitis and tumor formation. (**A**) Representative images of the back skin and tail of 8 to 10 weeks old OTULIN^FL/FL^, Δ^Ker^OTULIN, and Δ^Ker^OTULIN ΔNβcat mice. (**B**) Representative images of H&E-stained skin sections of 10-week-old mice of the indicated genotypes. Scale bar, 200 μm. (**C**) Time-course TEWL measurements on lesional skin of Δ^Ker^OTULIN and Δ^Ker^OTULIN ΔNβcat mice (*n* > 4 mice per condition, multiple lesions per mouse were scored). Data are means ± SEM, ^∗^*P* < 0.05; ^∗∗^*P* < 0.01; ^∗∗∗^*P* < 0.001; ****P < 0.0001. (**D**) Clinical scoring (see Materials and Methods) of back and tail skin inflammation in mice of the indicated genotypes at week 14 of age. Data represent means ± SEM, ****P < 0.0001. (**E**) Levels of IL-6 in serum of 10- to 14-week-old mice of the indicated genotypes (*n* > 4 mice per genotype) (*P < 0.05; ^∗∗^*P* < 0.01, Kruskal-Wallis test). (**F**) Immunostaining of skin sections from 10- to 14-week-old mice of the indicated genotypes using antibodies against cleaved caspase-3 (CC3), Ki67, TUNEL, K10, and K14. Each staining was performed on five mice per condition. Scale bar, 200 μm. (**G**) Quantification of the number of interfollicular epidermis (IFE) cells that stain positive for cleaved caspase-3 in skin sections from 10- to 14-week-old mice of the indicated genotypes (OTULIN^FL/FL^, *n* = 5; OTULIN^FL/FL^ ΔNβcat, *n* = 6; Δ^Ker^OTULIN NL, *n* = 4; Δ^Ker^OTULIN L, *n* = 6; Δ^Ker^OTULIN ΔNβcat NL, *n* = 6; Δ^Ker^OTULIN ΔNβcat L, *n* = 6). Data represent means ± SEM, *P < 0.05; **P < 0.01; ***P < 0.001.

Histomorphological analyses of the back skin revealed reduced epidermal hyperplasia in lesional skin of ∆^Ker^OTULIN ΔNβcat relative to ∆^Ker^OTULIN mice (Fig. 2F and fig. S2C), which was further confirmed by reduced Ki67 staining (Fig. 2F). Given that skin pathology associated with defective linear (de)ubiquitination in KCs is driven by excessive cell death (*12*, *13*), we next questioned whether stabilization of β-catenin protects OTULIN-deficient KCs from death. In lesional skin sections, a significant reduction in apoptotic interfollicular epidermal cells was observed in ∆^Ker^OTULIN ΔNβcat skin compared to ∆^Ker^OTULIN skin, as assessed by cleaved caspase-3 staining (Fig. 2, F and G). Similar reductions in cell death levels were observed using TUNEL (terminal deoxynucleotidyl transferase dUTP nick end labeling) staining, which marks both apoptotic and necroptotic cells (Fig. 2F). Immunostaining for keratin-10 (K10) and K14 revealed aberrant K14 expression in lesional skin of both ∆^Ker^OTULIN ΔNβcat and ∆^Ker^OTULIN mice (Fig. 2F), indicating that KC differentiation is impaired in lesional skin irrespective of the levels of β-catenin. OTULIN stabilizes LUBAC components in various cell-types, including KCs (*11*, *12*, *14*). One potential explanation for the marked improvement in the inflammatory phenotype of ∆^Ker^OTULIN mice upon β-catenin stabilization is that LUBAC proteins might also be stabilized under these conditions. However, immunoblotting for HOIL-1 and HOIP in epidermal lysates from ∆^Ker^OTULIN ΔNβcat and ∆^Ker^OTULIN mice showed a similar reduction in protein expression in both genotypes (fig. S2D). Multiple reports suggest a role for Wnt signaling in skin wound healing (*31*, *32*). When we subjected ∆^Ker^OTULIN and ∆^Ker^OTULIN ΔNβcat mice to full-thickness excisional skin wounding, we observed that ∆^Ker^OTULIN ΔNβcat mice close their wounds faster compared to ∆^Ker^OTULIN mice, as evidenced by accelerated epidermal barrier recovery (fig. S2E). Collectively, our findings demonstrate that skin inflammation driven by aberrant linear ubiquitination can be effectively mitigated in vivo by induction of canonical Wnt signaling.

### Therapeutic stabilization of β-catenin ameliorates dermatitis in ∆^Ker^OTULIN mice

To assess whether inducing Wnt signaling can halt the progression of established skin lesions, ∆^Ker^OTULIN ΔNβcat and ∆^Ker^OTULIN mice were injected with tamoxifen at 3 and 4 weeks of age, a time point when lesions were already present. Macroscopic examination of the skin revealed that inflammatory parameters, such as edema and erythema, either remained static or improved over time in ∆^Ker^OTULIN ΔNβcat mice, in contrast to the progressive inflammation observed in ∆^Ker^OTULIN skin (Fig. 3, A and B). Clinical scoring confirmed the beneficial effect of Wnt induction in lesional ∆^Ker^OTULIN skin, reducing lesion progression and tail skin involvement (Fig. 3C). Time course analysis of TEWL further demonstrated that stabilization of β-catenin prevents worsening of barrier perturbation (Fig. 3D). Moreover, cleaved caspase-3 and TUNEL staining indicated reduced cell death levels in lesional skin of ∆^Ker^OTULIN ΔNβcat mice relative to ∆^Ker^OTULIN mice (Fig. 3, E and F). These data show that promoting canonical Wnt signaling in KCs effectively halts the progression of skin inflammation caused by aberrant linear deubiquitination.

**Fig. 3.**
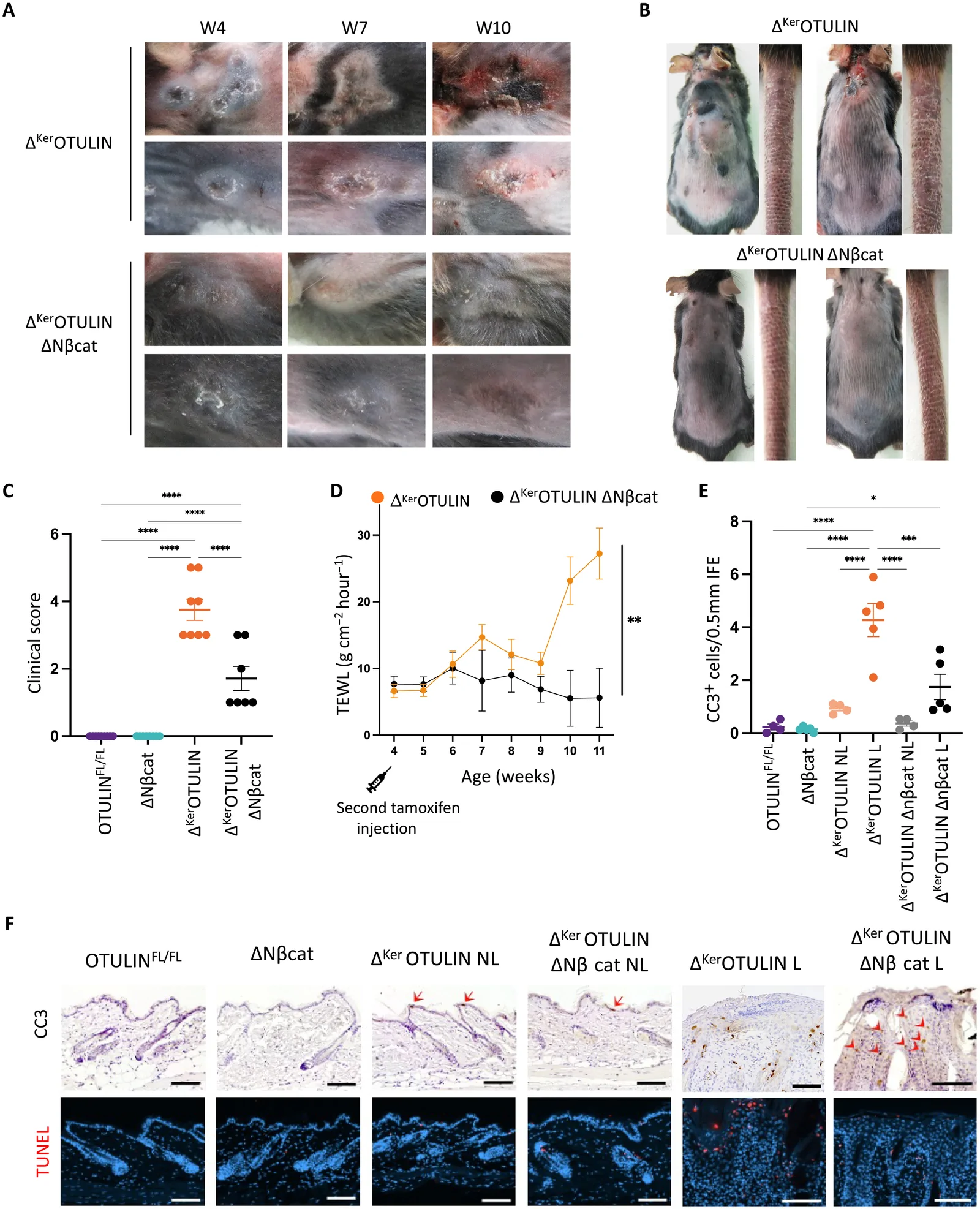
Stabilizing β-catenin in established Δ^Ker^OTULIN skin lesions prevents progressive inflammation. (**A**) Representative images of lesional back skin of Δ^Ker^OTULIN and Δ^Ker^OTULIN ΔNβcat mice at 4, 7, and 10 weeks of age (W). Mice were treated with tamoxifen at P21 and P28. (**B**) Representative images of the back skin and tail of 8- to 10-week-old Δ^Ker^OTULIN and Δ^Ker^OTULIN ΔNβcat mice treated with tamoxifen at P21 and P28. (**C**) Clinical scoring of back and tail skin inflammation in mice of the indicated genotypes at week 14 of age. Data represent means ± SEM, ****P < 0.0001. (**D**) Time-course TEWL measurements on lesional skin of Δ^Ker^OTULIN and Δ^Ker^OTULIN ΔNβcat mice (*n* > 4 mice per condition, multiple lesions per mouse were scored, **P < 0.01). (**E**) Quantification of the number of IFE cells that stain for cleaved caspase-3 in skin sections from 10- to 14-week-old mice of the indicated genotypes (OTULIN^FL/FL^, *n* = 4; OTULIN^FL/FL^ ΔNβcat, *n* = 4; Δ^Ker^OTULIN NL, *n* = 4; Δ^Ker^OTULIN L, *n* = 5; Δ^Ker^OTULIN ΔNβcat NL, *n* = 4; Δ^Ker^OTULIN ΔNβcat L, *n* = 5). Data represent means ± SEM, *P < 0.5; ***P < 0.001; ****P < 0.0001. (**F**) Immunostaining of skin sections from 10- to 14-week-old mice of the indicated genotypes using antibody against cleaved caspase-3 and TUNEL staining. Each staining was performed on *n* = 5 mice per condition. Scale bar, 200 μm. Arrows indicate apoptotic cells.

### OTULIN promotes β-catenin stability

Wnt/β-catenin activation was previously shown to be regulated by ubiquitination (*23*–*26*). To assess whether OTULIN mediates the stability of β-catenin in primary mouse KCs, we performed a K48-linked Ubq chain pulldown under denaturing conditions to precipitate proteins targeted for proteasomal degradation. In untreated conditions, a more substantial enrichment for β-catenin was observed in OTULIN-deficient KCs compared to control KCs upon K48 Ubq pulldown, indicating that proteasomal targeting of β-catenin is enhanced in absence of OTULIN (Fig. 4A). In KCs treated with the proteasome inhibitor MG132, smearing of β-catenin upon K48 pulldown became apparent in both control and OTULIN-deficient KCs. Immunoblotting for M1 Ubq chains in K48 Ubq pulldown lysates indicated an accumulation of proteins that are M1 and K48 ubiquitinated in OTULIN-deficient KCs relative to control KCs (Fig. 4A). Next, we performed a cycloheximide (CHX) chase for β-catenin in WNT3A-pretreated OTULIN-deficient and control mouse KCs. Monitoring β-catenin protein levels over time revealed faster degradation of β-catenin in the absence of OTULIN (Fig. 4, B and C). To investigate the role of linear deubiquitination in human KCs, we generated HaCaTs with strongly reduced OTULIN levels using OTULIN-targeting nanoblades (fig. S3, A and B). OTULIN knockdown (KD) HaCaT cells displayed increased sensitivity to TNF-induced cell death, consistent with our previous observations in OTULIN-deficient mouse KCs (fig. S3C) (*12*). Analysis of an interaction between β-catenin and OTULIN in human KCs was carried out through coimmunoprecipitation (IP) studies using a β-catenin–specific antibody. Pulldown analysis indicated that β-catenin and OTULIN coprecipitate (Fig. 4D and fig. S3, D and E). Incubation of β-catenin IP lysates from OTULIN KD and control HaCaT cells with recombinant human OTULIN resulted in the collapse of the M1-linked Ubq smearing on both unphosphorylated and total β-catenin, corroborating its linear Ubq status in human KCs (Fig. 4E). Collectively, these findings demonstrate that OTULIN interacts with β-catenin in homeostatic KCs, modulating its linear Ubq status and promoting its stability.

**Fig. 4.**
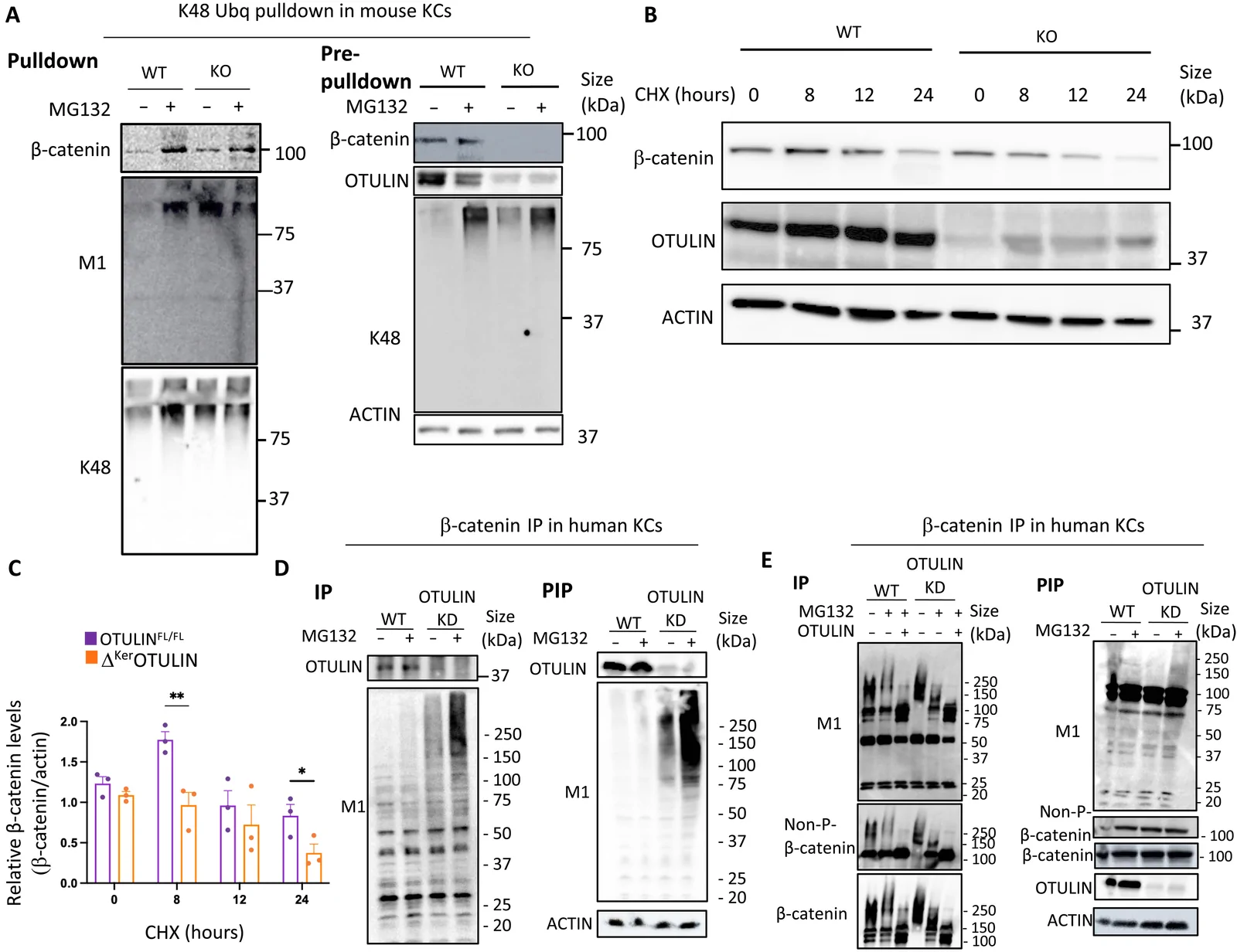
β-catenin is linearly ubiquitinated and coprecipitates with OTULIN in adult KCs. (**A**) K48 Ubq pulldown (left) and corresponding pre-pulldown (right) lysates of primary mouse KCs isolated from OTULIN^FL/FL^ and ∆^Ker^OTULIN mice pretreated or not with MG132 for 16 hours and immunoblotted for β-catenin, OTULIN, K48 Ubq, or M1 Ubq chains. α-Actin is shown as loading control. (**B**) Primary mouse KCs isolated from OTULIN^FL/FL^, and ∆^Ker^OTULIN mice were pretreated with WNT3A (50 ng/ml) for 24 hours and subsequently exposed to CHX (20 μg/ml) for the indicated time. Lysates were immunoblotted for β-catenin and OTULIN. α-Actin is shown as loading control. Immunoblots are representative of three independent experiments. (**C**) Densitometric analyses of ratio of β-catenin to actin in untreated and CHX-treated KCs (two-way ANOVA with multiple comparisons, **P = 0.0045; *P = 0.0173). (**D**) Co-IP using an antibody against β-catenin on lysates from WT and OTULIN KD HaCaTs left untreated or treated with MG132 for 16 hours. Preimmunoprecipitation (PIP) lysates were blotted for the same proteins, and α-actin is shown as loading control. (**E**) Immunoprecipitation lysates using an antibody against β-catenin were untreated or supplemented with recombinant human OTULIN post-IP, followed by immunoblotting for M1 Ubq, active β-catenin, and total β-catenin.

### OTULIN-dependent Wnt signaling protects KCs from death

We and others previously demonstrated that skin inflammation in ∆^Ker^OTULIN mice is driven by TNF-mediated apoptotic and necroptotic KC death (*12*, *13*). To investigate whether the enhanced sensitivity of OTULIN-deficient KCs to TNF-induced cell death is normalized upon β-catenin stabilization, primary KCs isolated from ∆^Ker^OTULIN, ∆^Ker^OTULIN ΔNβcat, and control mice were stimulated with TNF following pretreatment with interferon γ (IFNγ). IFNγ pretreatment was used to assess TNF cytotoxicity under conditions of ZBP1 (Z-DNA binding protein 1) up-regulation, as OTULIN deficiency in mouse KCs is known to induce IFN responses (*12*, *33*). OTULIN-deficient KCs with stabilized β-catenin were markedly protected from TNF-induced apoptosis, as evidenced by decreased cleaved caspase-3 levels compared with ∆^Ker^OTULIN KCs (Fig. 5A). In agreement, reduced levels of cleaved caspase-8 and cleaved caspase-3 were detected in tail whole mounts from ∆^Ker^OTULIN ΔNβcat compared to ∆^Ker^OTULIN epidermis (Fig. 5, B and C). Protection from TNF-induced cell death was further confirmed in primary mouse KCs from ∆^Ker^OTULIN ΔNβcat mice relative to KCs from ∆^Ker^OTULIN mice using dye penetration assays (Fig. 5D). To analyze whether the increased KC cell death levels repress Wnt signaling in ∆^Ker^OTULIN skin, we quantified Wnt target gene expression in ∆^Ker^OTULIN/FADD/MLKL mice, which are protected from FADD and MLKL-dependent KC death and subsequent skin inflammation (*12*). qPCR analyses indicated that the levels of various Wnt mediators and target genes were similarly reduced in both ∆^Ker^OTULIN/FADD/MLKL and ∆^Ker^OTULIN skin (Fig. 5E), indicating that OTULIN promotes Wnt signaling independently of KC cell death levels. Last, pretreatment of KCs with WNT3A reduced TNF-induced cell death in OTULIN-deficient cells (Fig. 5F). Collectively, these findings indicate that the induction of Wnt signaling by OTULIN sets the threshold for KC susceptibility to TNF-induced cell death.

**Fig. 5.**
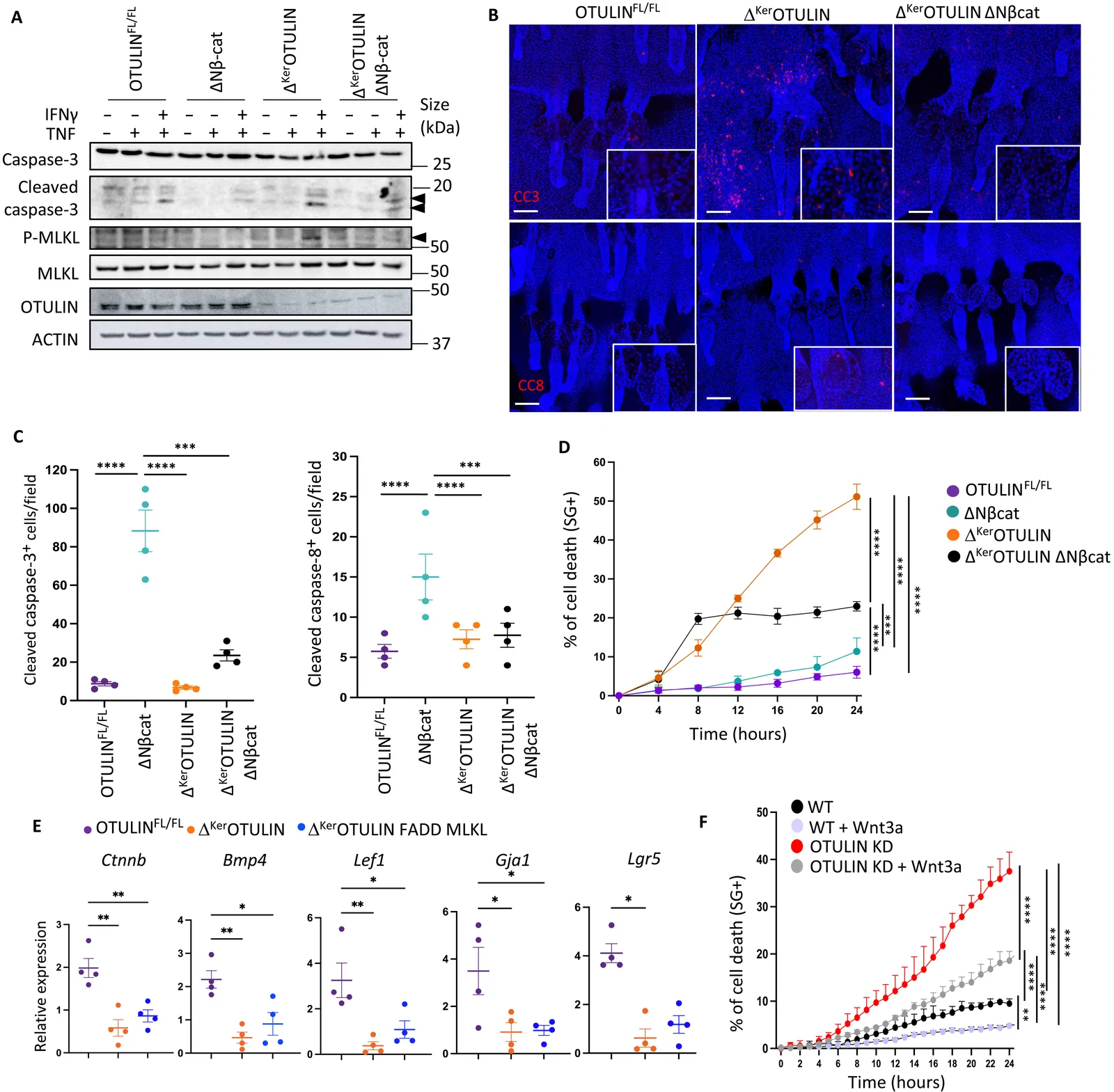
OTULIN-driven Wnt signaling protects KCs from death. (**A**) Immunoblotting for (cleaved) caspase-3 of lysates from KCs isolated from OTULIN^FL/FL^ and ∆^Ker^OTULIN mice. Cells were untreated or pretreated overnight with IFNγ prior to TNF treatment. Actin blot is shown as loading control. Arrowheads indicate cleavage fragments of caspase-3. (**B**) Immunofluorescence staining for cleaved caspase-3 and cleaved caspase-8 on epidermal tail whole mounts from OTULIN^FL/FL^, ∆^Ker^OTULIN, and Δ^Ker^OTULIN ΔNβcat mice. Insets represent magnified views. Scale bars, 100 μm. (**C**) Quantification of cleaved caspase-3 (left) and cleaved caspase-8–positive (right) cells per field (*n* = 4 mice per genotype; one-way ANOVA). Data represent means ± SEM of three different microscopic fields. (**D**) Viability assay on primary mouse KCs isolated from OTULIN^FL/FL^, ΔNβcat, ∆^Ker^OTULIN mice, and Δ^Ker^OTULIN ΔNβcat mice pretreated with IFNγ and treated for 24 hours with TNF. Viability was assessed by SYTOX Green (SG) uptake on Incucyte images (two-way ANOVA with multiple comparisons, ***P < 0.001; ****P < 0.0001). (**E**) Relative mRNA expression of the indicated Wnt target genes in epidermal tail lysates of OTULIN^FL/FL^, ∆^Ker^OTULIN, and Δ^Ker^OTULIN FADD MLKL mice. Data represent means ± SEM (*P < 0.05; **P < 0.01; ***P < 0.001). (**F**) Viability assay on WT and OTULIN KD HaCaTs pretreated for 24 hours with WNT3A (50 ng/ml) and subsequently treated with for 24 hours with TNF. Viability was assessed by SYTOX Green uptake on Incucyte images (two-way ANOVA with multiple comparisons, **P < 0.01; ****P < 0.0001).

### OTULIN regulates the stability of the survival factors TCF3 and TCF4

To investigate how linear ubiquitination of β-catenin influences KC survival, we focused on two Wnt target genes, *Tcf3* and *Tcf4*, previously shown to protect cells from death by cooperative functioning with β-catenin to drive transcription (*22*, *34*, *35*). *Tcf3* and *Tcf4* were significantly suppressed in OTULIN-deficient mouse KCs (Fig. 1D). Previous studies demonstrated that genetic ablation of *Tcf3/4* in KCs results in a marked increase of dying cells in neonatal mouse epidermis (*34*). Immunoblotting for TCF3 revealed a reduction in protein expression in OTULIN-deficient primary mouse KCs relative to WT KCs (Fig. 6A). Proteasome inhibition stabilized TCF3 in both WT and OTULIN-deficient cells, indicating that TCF3 stability is regulated by K48-linked ubiquitination in KCs. Both Wnt-repressive and Wnt-activating properties have been attributed to TCF3/4 (*34*). TCF3 expression in primary KCs strongly correlated with the levels of activated β-catenin levels, suggesting that TCF3 functions as a Wnt activator under these conditions (Fig. 6A). Consistently, *Tcf3* and *Tcf4* levels were reduced in epidermal lysates obtained from ∆^Ker^OTULIN skin relative to control epidermis, and no significant difference in *Tcf3* or *Tcf4* levels was observed between control epidermis and epidermis from ∆^Ker^OTULIN ΔNβcat mice (Fig. 6B). These findings indicate that the protection from cell death observed in KCs from ∆^Ker^OTULIN ΔNβcat mice relative to ∆^Ker^OTULIN mice could be linked to altered TCF3/4 expression levels. To test this hypothesis, OTULIN KD HaCaTs were reconstituted with TCF3 by lentiviral transduction (fig. S4A). Reconstitution of TCF3 significantly reduced TNF cytotoxicity in OTULIN KD cells; however, cell death in these reconstituted KCs remained higher than in control KCs (Fig. 6C). This indicates that TCF3/4 contribute to the cellular response to TNF, with evidence suggesting a modulatory, rather than exclusively protective, role in cell viability. Immunofluorescence staining for TCF4 in tail whole mounts of control mice confirmed TCF4 expression in adult normal HFs (*36*), whereas no TCF4 could be observed in ∆^Ker^OTULIN skin (Fig. 6D). In contrast, TCF4 expression in the tail epidermis of ∆^Ker^OTULIN ΔNβcat mice was comparable to that observed in control mice. Notably, co-staining for TCF4 with cleaved caspase-3 revealed that TCF4-expressing KCs were not undergoing apoptosis, as no double-stained cells were observed (Fig. 6D).

**Fig. 6.**
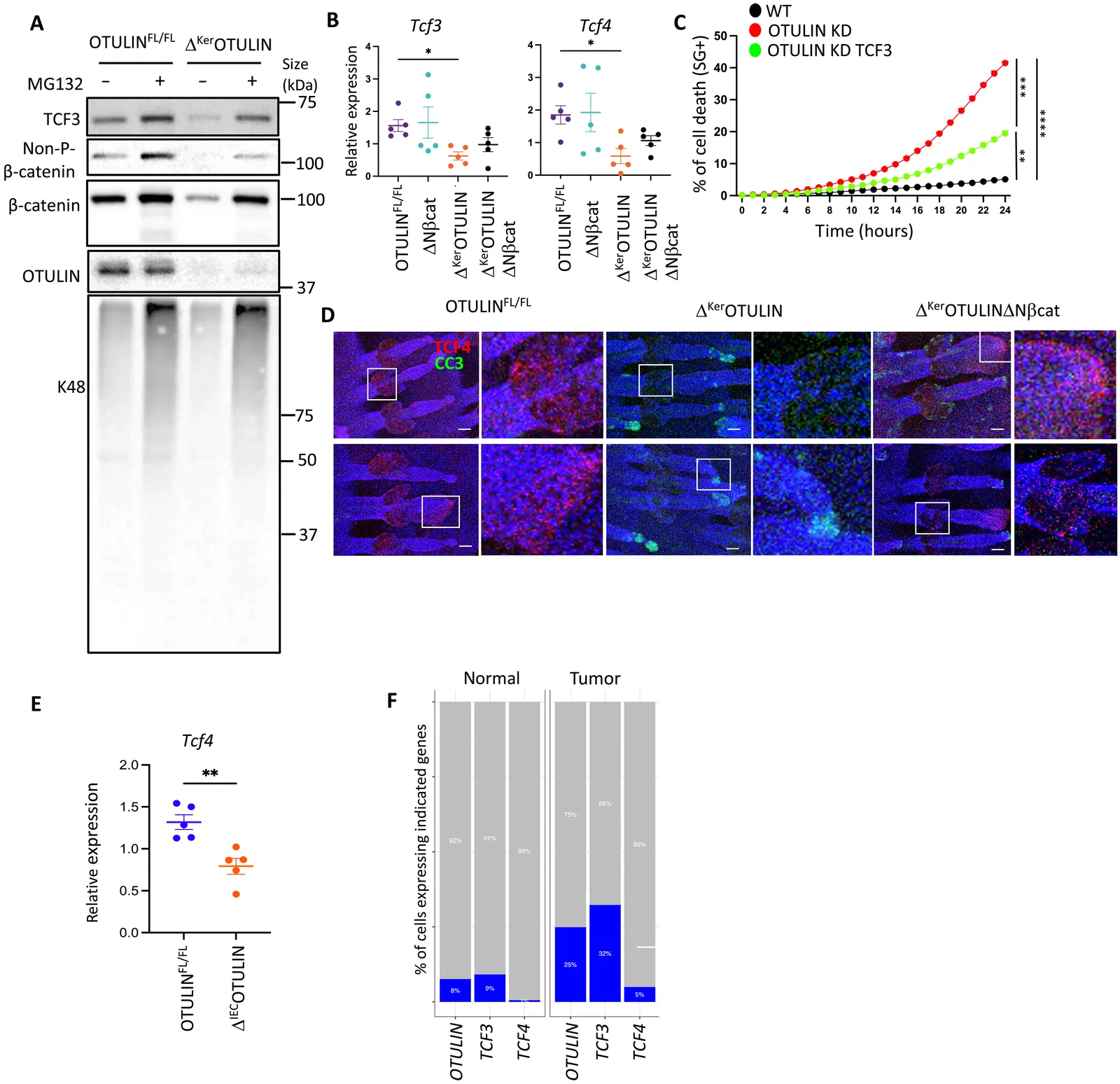
OTULIN promotes TCF3/4 stability. (**A**) Western blot analysis of lysates from untreated or MG132-treated primary mouse KCs isolated from OTULIN^FL/FL^ and ∆^Ker^OTULIN mice using antibodies against TCF3, β-catenin, nonphosphorylated β-catenin, OTULIN, and K48 Ubq. (**B**) Relative mRNA expression of *Tcf3* and *Tcf4* in epidermal tail lysates of OTULIN^FL/FL^, ΔNβcat, ∆^Ker^OTULIN, and Δ^Ker^OTULIN ΔNβcat mice. Data represent means ± SEM (*P < 0.05). (**C**) Viability assay on WT, OTULIN KD, and OTULIN KD HaCaTs reconstituted with TCF3. Cells were treated with for 24 hours with TNF. Viability was assessed by SYTOX Green uptake on Incucyte images (two-way ANOVA with multiple comparisons, **P < 0.01; ***P < 0.001; ****P < 0.0001). (**D**) Immunofluorescence staining for TCF4 (red) and cleaved caspase-3 (green) in epidermal tail whole mounts of OTULIN^FL/FL^, ∆^Ker^OTULIN, and Δ^Ker^OTULIN ΔNβcat mice. Scale bars, 50 μm. White boxes indicate higher magnification insets. (**E**) Relative mRNA expression of *Tcf4* in intestinal lysates of OTULIN^FL/FL^ and ∆^IEC^OTULIN mice. Data represent means ± SEM (*P* = 0.0035). (**F**) Percentage of epithelial cells expressing OTULIN, TCF3, or TCF4 in normal human colon tissue versus colorectal cancer (tumor) samples as observed by scRNA-seq (*39*).

Last, we questioned whether OTULIN can drive canonical Wnt signaling in tissues beyond the skin. TCF3 and TCF4 are transcription factors associated with repression of differentiation, not only in skin, but also in intestinal epithelia (*35*, *37*). Analysis of *Tcf4* levels in intestinal epithelial cells (IECs) isolated from IEC-selective OTULIN-deficient and WT mice confirmed a significant down-regulation of *Tcf4* in OTULIN-deficient IECs (Fig. 6E). Supporting a role for OTULIN in regulating stemness, our previously published scRNA-seq dataset comparing control and ∆^Ker^OTULIN nonlesional (NL) and lesional (L) skin (*12*) revealed higher expression of the HF stem cell markers *Cd34* and *Lgr5* in OTULIN-positive versus OTULIN-negative KCs (fig. S4B). Given the frequent dysregulation of Wnt signaling in cancer and the dependence of various tumor types on canonical Wnt activation for growth and progression (*22*), these findings suggest a broader role for OTULIN in modulating Wnt-driven processes, as also suggested by others (*26*). Colorectal cancer is a well-characterized Wnt-driven tumor type, often promoted by mutations in Wnt pathway genes such as *APC*, *CTNNB1*, and *AXIN2* (*38*). Analysis of a publicly available scRNA-seq dataset of human colorectal cancer samples and healthy colon tissue (Human Colon Cancer Atlas, GEO: GSE178341) (*39*) revealed elevated levels of *OTULIN, TCF3*, and *TCF4* in epithelial tumor cells compared with normal IECs (Fig. 6F and fig. S4C). Together, these findings highlight OTULIN as a driver of Wnt signaling across various epithelial tissues and disease contexts.

## DISCUSSION

Protection from cell death is crucial for KCs to ensure proper epidermal barrier function. The physiological impact of KC death depends on both the mode of cell death and the spatial context of the tissue (*40*). Aberrant linear deubiquitination due to absence of OTULIN predisposes KCs to apoptosis and necroptosis, two modes of regulated cell death that contribute to the development of dermatitis and squamous carcinogenesis (*12*, *13*, *41*). In the original study identifying OTULIN as the DUB for linear Ubq chains, a potential role for OTULIN in promoting canonical Wnt signaling in endothelial cells was already proposed (*17*). However, direct physiological evidence supporting this connection has been lacking. Overexpression studies later suggested that SHARPIN can regulate Wnt signaling independently of linear ubiquitination by binding with β-catenin and thereby preventing its association with β-TrCP1 (*28*). In this study, we provide physiological evidence positioning OTULIN as a dual-function survival factor in KCs. We show that OTULIN’s DUB activity not only stabilizes LUBAC to prevent TNF-induced cell death and inflammation but also promotes canonical Wnt signaling in KCs. Notably, inhibition of cell death by depletion of cell death executioners in OTULIN-deficient KCs did not restore Wnt target gene expression, indicating that Wnt down-regulation occurs independently of KC death. Moreover, stabilization of β-catenin in KCs, through genetic crossing with mice expressing a truncated variant of β-catenin, potently halted or prevented the progressive dermatitis caused by OTULIN deficiency. This truncated β-catenin lacks lysine residues required for K48-linked ubiquitination, as well as K133, the residue implicated in linear ubiquitination by LUBAC (*26*). Induction of truncated β-catenin expression in KCs of ∆^Ker^OTULIN mice significantly reduced KC death and subsequent skin inflammation. The prosurvival effects of β-catenin stabilization were independent of LUBAC protein levels, which remained down-regulated in ∆^Ker^OTULIN ΔNβcat mice. These data indicate that canonical Wnt signaling can, at least partially, bypass cell death checkpoints controlled by LUBAC activity (*42*). Notably, primary KCs from ∆^Ker^OTULIN ΔNβcat mice exhibited reduced TNF-induced cell death relative to KCs from ∆^Ker^OTULIN mice, suggesting that the extent of canonical Wnt signaling sets the threshold for TNF cytotoxicity in OTULIN-deficient KCs. Furthermore, we observed comparable down-regulation of canonical Wnt signaling in OTULIN-deficient and SHARPIN^cpdm/cpdm^ epidermis, underscoring the pivotal role of linear (de)ubiquitination in fine-tuning canonical Wnt pathway activity.

Our findings indicate that β-catenin is linearly ubiquitinated in homeostatic KCs. In the absence of OTULIN, β-catenin stability is reduced due to its enhanced K48-linked ubiquitination and subsequent proteasomal degradation. Notably, smearing of β-catenin, indicative of ubiquitination, was only observed in pulldown assays. This could be explained by the presence of multiple β-catenin pools within KCs in which only a fraction of β-catenin molecules is linearly and/or K48-ubiquitinated. Consistent with this, β-catenin localized at adherens junctions may be protected from ubiquitination events, as canonical Wnt signaling is mediated by the cytoplasmic pool of β-catenin. Previous studies suggested a role for OTULIN in stabilizing β-catenin in breast cancer cell lines exposed to genotoxic stress (*26*). Our findings extend this concept, demonstrating that β-catenin undergoes linear ubiquitination in normal adult KCs, both in vivo and in vitro. In the absence of OTULIN, β-catenin exhibits increased K48-linked ubiquitination, resulting in its proteasomal degradation. We establish that this regulatory mechanism operates in homeostatic KCs, where β-catenin interacts with OTULIN to modulate KC susceptibility to cell death. In agreement, a study probing for linearly ubiquitinated substrates in glioblastoma cells identified β-catenin as an M1 ubiquitination target (*43*). However, it remains unclear whether OTULIN-mediated regulation of Wnt signaling represents a general mechanism for safeguarding cell viability in conditions of stress.

The levels of activated β-catenin closely correlated with TCF3 expression in KCs, and both were markedly reduced in absence of OTULIN. TCF3 and TCF4 function as transcription factors upon binding with β-catenin (*35*) and are essential regulators of postnatal KC survival (*34*). We show that TCF3/4 stability appears to depend on OTULIN expression. Notably, TCF3 and TCF4 are known repressors of differentiation in postnatal skin and IECs (*34*, *36*, *37*). In OTULIN-deficient KCs, TCF3 and TCF4 are markedly down-regulated, potentially opposing differentiation of stem cells into HF cells. This is supported by our scRNA-seq data, which reveal a marked increase in the relative abundance of HF bulge cells in ∆^Ker^OTULIN skin. In IECs, TCF3/4 levels are down-regulated in adult tissues but are re-expressed in epithelial colorectal tumor cells (*37*). This re-expression is associated with a more undifferentiated state and coincides with OTULIN up-regulation. In line with these observations, we found that reconstitution of TCF3 in OTULIN KD KCs reduced cytotoxicity of TNF. Together, these findings indicate that OTULIN, by driving canonical Wnt signaling and maintaining TCF3/4 expression, may constitute a survival pathway in various cell types and disease contexts.

In conclusion, our findings establish OTULIN as a critical regulator of KC survival through a dual mechanism: mediating the linear (de)ubiquitination of proteins involved in the TNF pathway and promoting canonical Wnt signaling upstream of cell death. This two-tiered mechanism likely acts as a safeguard to maintain stemness and suppress differentiation in conditions of heightened proliferative needs, as is the case in inflammation.

## MATERIALS AND METHODS

### Mice

The following mouse lines were used: *Otulin^FL^* (*44*), *K14-Cre* (*45*), *Fadd^FL^* (*46*), *Mlkl^FL^* (*47*), *SHARPIN^cpdm^* (*48*), Δ*N*β*cateninER*^*Ki*/+^ line D2 (*30*). All alleles were maintained on a C57BL/6 genetic background. Mice were housed in individually ventilated cages at the VIB Center for Inflammation Research, in a specific pathogen-free animal facility. All animal experiments were conducted according to institutional, national, and European animal regulations for animal testing and research. Animal protocols were approved by the VIB-Ghent University ethical review board (study approval numbers: LP0035, EC2024-008, and EC2024-009). Animals requiring medical attention were provided with appropriate care and were sacrificed when reaching predetermined criteria of disease severity. Female and male mice of the indicated genotype were assigned at random to groups. Mouse studies as well as microscopy-based assessment of pathology were performed in a blinded fashion. Inducible mouse lines were treated topically with 4-hydroxytamoxifen (20 mg/ml) at P0.5 and P1.5 or by intraperitoneal injection of tamoxifen (10 mg/ml) at older age.

### Clinical scoring of lesions

Mice were scored for clinical signs of inflammation: A score of 0 to 3 was assigned to each mouse based on the size of lesions on the back skin: 0 = no lesions; 1 = all skin lesions smaller than 3 mm^2^; 2 = at least one skin lesions bigger than 3 mm^2^; 3 = papilloma-like structures. Added to this score was a score of 0 to 2 based on the level of tail skin inflammation: 0 = normal; 1 = inflammation; 2 = loss of tail caused by severe inflammation, totaling up to a score of 0 to 5 to assess skin inflammation.

### KC cultures

Primary mouse KCs were isolated from OTULIN^FL/FL^, Δ^Ker^OTULIN, OTULIN^FL/FL^ ΔNβcat, and Δ^Ker^OTULIN ΔNβcat skin as previously described (*49*). Briefly, shaved back skin was isolated, sterilized, and floated on 0.25% trypsin overnight. The epidermis was separated from dermis and cultured on confluent feeder cultures. Primary mouse KCs were cultured in FAD medium (Bio&Sell) containing one part Ham’s F12, three parts Dulbecco’s modified Eagle’s medium, 0.18 mM adenine, supplemented with 10% chelated fetal calf serum (Bodinco, S-FBS-EU-015) and a cocktail of hydrocortisone (0.5 μg/ml; Sigma-Aldrich, H4001), insulin (5 μg/ml; Sigma-Aldrich, I3536), and 1 × 10^−10^ M cholera enterotoxin (Sigma-Aldrich, C8052). Murine cells were pretreated for 8 to 12 hours with IFNγ (10 ng/ml; VIB Protein Service Facility) and subsequently stimulated with murine TNF (30 ng/ml; VIB Protein Service Facility) for 12 hours, or treated with MG132 (0.25 μM; Sigma-Aldrich 474790) for 12 to 16 hours. Generation of HaCaTs with KD of OTULIN was achieved using virus-like particles (nanoblades). Two single guide RNAs were used to create a 29–base pair deletion in the *OTULIN* gene. HaCaTs were treated with 20 pM nanoblades for 24 hours for two consecutive rounds, followed by TIDE analysis. HaCaTs with a predicted indel efficiency of over 95% were used for further experimentation. WT and OTULIN KD HaCaTs were stimulated with human TNF (30 ng/ml; VIB Protein Service Facility) for 12 hours or treated with MG132 (0.25 μM; Sigma-Aldrich, 474790) for 12 to 16 hours.

### Cell death assays

Cell death assays were performed on subconfluent KCs in six-well plates. Cells were pretreated as indicated in figure legends and stimulated with TNF (30 ng/ml) in the presence of 2.5 μM SYTOX Green Nucleic Acid Stain (S7020, Molecular Probes). SYTOX Green uptake was imaged every 1 or 4 hours with an IncuCyteS3 Live-Cell Analysis System (Essen BioScience). The relative percentage of SYTOX Green–positive cells was determined by dividing the number of SYTOX Green–positive cells per image by the percentage of confluency using phase contrast images.

### Real-time qPCR

Total RNA was isolated using TRIzol reagent (Invitrogen) and Aurum Total RNA Isolation Mini Kit (Biorad), according to the manufacturer’s instructions. Synthesis of cDNA was performed using SensiFAST cDNA Synthesis Kit (Bioline). cDNA was amplified on qPCR in a total volume of 10 μl with SensiFAST SYBR No-ROX Kit (Bioline) and specific primers on a LightCycler 480 (Roche). The reactions were performed in technical triplicates. The mouse-specific primers used are summarized in Table 2.

### Lentiviral transduction of HaCaTs

Human embryonic kidney–293 cells were transfected using calcium phosphate, either with a vector coding for human TCF3 or the empty vector backbone (Addgene), and the viral packaging vectors pMD2-VSVG (in-house) and pCMVΔR8.91 (in-house). Six hours posttransfection, medium was changed. After 2 days, medium containing lentiviral particles was harvested and filtered (0.22 μm). HaCaTs were seeded at 1 × 10^5^ cells per well in a six-well plate. The following day, polybrene (8 μg/ml) was added to the culture medium, and a twofold dilution series of virus was made in the first five wells, with the last well serving as a nontransduced control. Plates were subsequently spun down for 1 hour at 800*g* and 32°C. Medium was added and after 2 days, and cells stably expressing the construct of interest were selected with puromycin (0.5 μg/ml).

### Western blotting

Primary KCs and epidermal tail tissue were homogenized using E1A lysis buffer (50 mM Hepes, pH 7.6; 250 mM NaCl; 5 mM EDTA; 0.5% NP40) or NP-40 lysis buffer (50 mM tris-HCl, pH 7.6; 1 mM EDTA; 150 mM NaCl; 1% NP-40; 0.5% sodiumdeoxycholate; 0.1% SDS), respectively containing protease inhibitors (Roche) and phosphatase inhibitors (Sigma-Aldrich). Epidermal or cell lysates were separated by SDS–polyacrylamide gel electrophoresis and transferred to nitrocellulose membranes. Membranes were probed with antibodies (Table 2) against OTULIN (1:1000, Cell Signaling Technology, 14127), HOIL-1 (1:2000, Henning Walczak laboratory), HOIP (1:1000, Rune Damgaard laboratory), SHARPIN (1:1000, Proteintech, 14626-1-AP), linear Ubq (1:2500, Sigma-Aldrich, ZRB2114), K48 Ubq (1:1000; Cell Signaling, D9D5), caspase-3 (1:1000; Cell Signaling, 9662), MLKL (1:1000, Millipore, MABC604), P-MLKL (1:1000, Abcam, ab196436), β-catenin (1:2000, BD, 610153), nonphosphorylated-β-catenin (1:1000, Merck, 05-665), TCF3 (1:1000, Cell Signaling, D15G11), and actin-HRP (1:10,000, Santa Cruz Biotechnology, SC-47778HRP). As secondary antibodies, HRP coupled anti-rabbit-HRP, anti-mouse-HRP, and anti-goat-HRP were used (1:2500, Amersham), and detection was done by chemiluminescence (Western Lightning Plus ECL, Perkin Elmer) using an Amersham Imager 600 (GE Healthcare).

### Precipitation studies

Cell lysates from primary mouse KCs were prepared under denaturing conditions using RIPA lysis buffer [150 mM NaCl, 1% NP-40, 0.5% sodium deoxycholate, 0.1% SDS, and 10 mM tris-HCl (pH 8) supplemented with phosphatase and protease inhibitor cocktail tablets (Roche Diagnostics)], protein concentration was determined, and equal concentrations of protein lysates were incubated overnight with K48 Ubq-binding beads (K48 TUBE HF, Lifesensors, UM607). The next day, the beads were washed three times in RIPA lysis buffer. Beads were then resuspended in 60 μl of 1× Laemmli buffer for direct analysis.

### Coimmunoprecipitation studies

Protein G Sepharose beads (Cytiva, 17-0618-01) were washed three times with NP-40 (50 mM tris-HCl, pH 7.6; 1 mM EDTA; 150 mM NaCl; 1% NP-40; 0.5% sodiumdeoxycholate; 0.1% SDS) buffer containing protease inhibitors (Roche) and phosphatase inhibitors (Sigma-Aldrich) before being coupled to anti-β-catenin (10 μl/ml; BD, 610153) overnight. Cell lysates from HaCaTs were prepared using NP-40 buffer. Protein concentration was determined, and equal concentrations of protein lysates were incubated overnight with β-catenin–coupled beads. The next day, the beads were washed three times in NP40 lysis buffer. Beads were then resuspended in 60 μl of 1× Laemmli buffer for direct analysis.

### Linear deubiquitination of cell lysates with recombinant OTULIN

Co-IP was performed as mentioned above. After the overnight incubation step with β-catenin–coupled beads, protein complexes were deubiquitinated by incubation of beads with 2 μg (1 μM) recombinant human OTULIN protein (Abcam, ab268829). Recombinant OTULIN was first diluted to 2 μM in DUB dilution buffer [25 mM tris (pH 7.5), 150 mM NaCl, and 10 mM dithiothreitol (DTT)] to achieve full activity through reduction of oxidized species. Subsequently recombinant OTULIN was diluted to the final concentration (1 μM) in DUB reaction buffer [50 mM tris-HCl (pH 8), 50 mM NaCl, 5 mM DTT, and 1 mM MnCl_2_] and added to the beads for 30 min at 37°C. Beads were washed one time with DUB reaction buffer, and proteins were eluted in 50 μl of 1× LL (3 min, 70°C).

### Cytokine detection

Cytokine concentrations in serum were determined by a magnetic bead–based multiplex assay using Luminex technology (Bio-Rad), according to the manufacturer’s instructions.

### Analysis of scRNA-seq data

The previously obtained scRNA-seq dataset (*12*) was probed for gene expression levels using Seurat version 5.0.3. Heatmaps, violin plots, and dotplots were generated utilizing Seurat’s default functions. GO enrichment analysis was performed using the enrichGO function from clusterProfiler (v4.12.6) package. The analysis was conducted on the down-regulated genes from NL versus WT KCs with a *P* value cutoff of 0.05. The publicly available scRNA-seq dataset GSE178341 (*39*) on human colon tissue was analyzed for normalized relative expression of *OTULIN, TCF3*, and *TCF4* in epithelial cells.

### Skin wounding

Full-thickness skin wounds were inflicted with an 8-mm punch biopsy on 7-week-old female and male mice after shaving of the back skin and subcutaneous administration of Vetergesics (15 μg/ml). Wound sizes were measured with electronic callipers every other day by two independent researchers who were blinded to the genotypes. Wounding procedures were repeated in three independent experiments with similar results and performed under analgesia and general anesthesia.

### Immunofluorescence

Dewaxed paraffin skin sections or tail whole mounts were labeled with respective antibodies listed in table S2. As secondary antibodies, donkey-anti-mouse AlexaFluor 488, donkey anti-rabbit DyLight 488, goat anti-mouse DyLight 633, and goat-anti-rabbit DyLight 586 were used (1:2000) in combination with 4′,6-diamidino-2-phenylindole (DAPI). TUNEL kit (Millipore) was used according to manufacturer’s instructions.

### Immunohistochemistry

Dewaxed paraffin skin sections were subjected to heat-mediated antigen retrieval (citrate buffer; pH = 6) and labeled with Ki67 Ab (1: 1000; Cell Signaling Technology, D3B5, 12202) or cleaved caspase-3 Ab (1: 500; Cell Signaling Technology, 9661). Next, the slides were incubated with biotin-coupled goat anti-rabbit (1:500, Dako). Subsequently, slides were incubated in ABC solution (Vectastain ELITE ABC Kit Standard, Vector Laboratories). Peroxidase activity was detected by adding diaminobutyric acid (DAB) substrate [ImmPACT DAB Peroxidase (HRP) Substrate, Vector Laboratories].

### TEWL measurement

Mice were shaved prior to TEWL assessment. TEWL was measured on the back skin of anesthetized mice using a TEWA meter (Courage and Khazaka, TM 210). Lesions were followed over time. If multiple lesions per mouse were present, these were all assessed.

### Statistics

Results are expressed as the means ± SEM, with *n* as the number of mice. Normality tests (Kolmogorov-Smirnov) were performed to check for normal distribution of the data. Parametric tests were performed on normally distributed data, and nonparametric tests on not normally distributed data. Statistical significance of data obtained from different genotypes was assessed using two-way analysis of variance (ANOVA) tests, unpaired two-sided Student’s *t* tests or nonparametric Mann-Whitney tests, or one-way ANOVA test followed by Tukey’s multiple comparisons test. The analysis was performed with Prism 9 software. Time-course analysis of TEWL in Fig. 3D and cell death kinetics in fig. S3C were analyzed as repeated measurements using method of residual maximum likelihood (REML), as implemented in Genstat version 21.
