# Supplementary material for: Canonical Wnt induction by OTULIN prevents keratinocyte death and skin inflammation
Source: Sci Adv. 2026 Oct 9;12(41):eadz0181. doi: 10.1126/sciadv.adz0181 (PMC13651886; doi:10.1126/sciadv.adz0181)
Supplement: Supplementary file 1 [file sciadv.adz0181_sm.pdf]

Science Advances
AAAS

Supplementary Materials for

Canonical Wnt induction by OTULIN prevents keratinocyte death and skin inflammation

Kim Lecomte *et al.*

Corresponding author: Esther Hoste, esther.hoste@irc.vib-ugent.be

Sci. Adv. **12**, eadz0181 (2026)
DOI: 10.1126/sciadv.adz0181

This PDF file includes:

Figs. S1 to S4

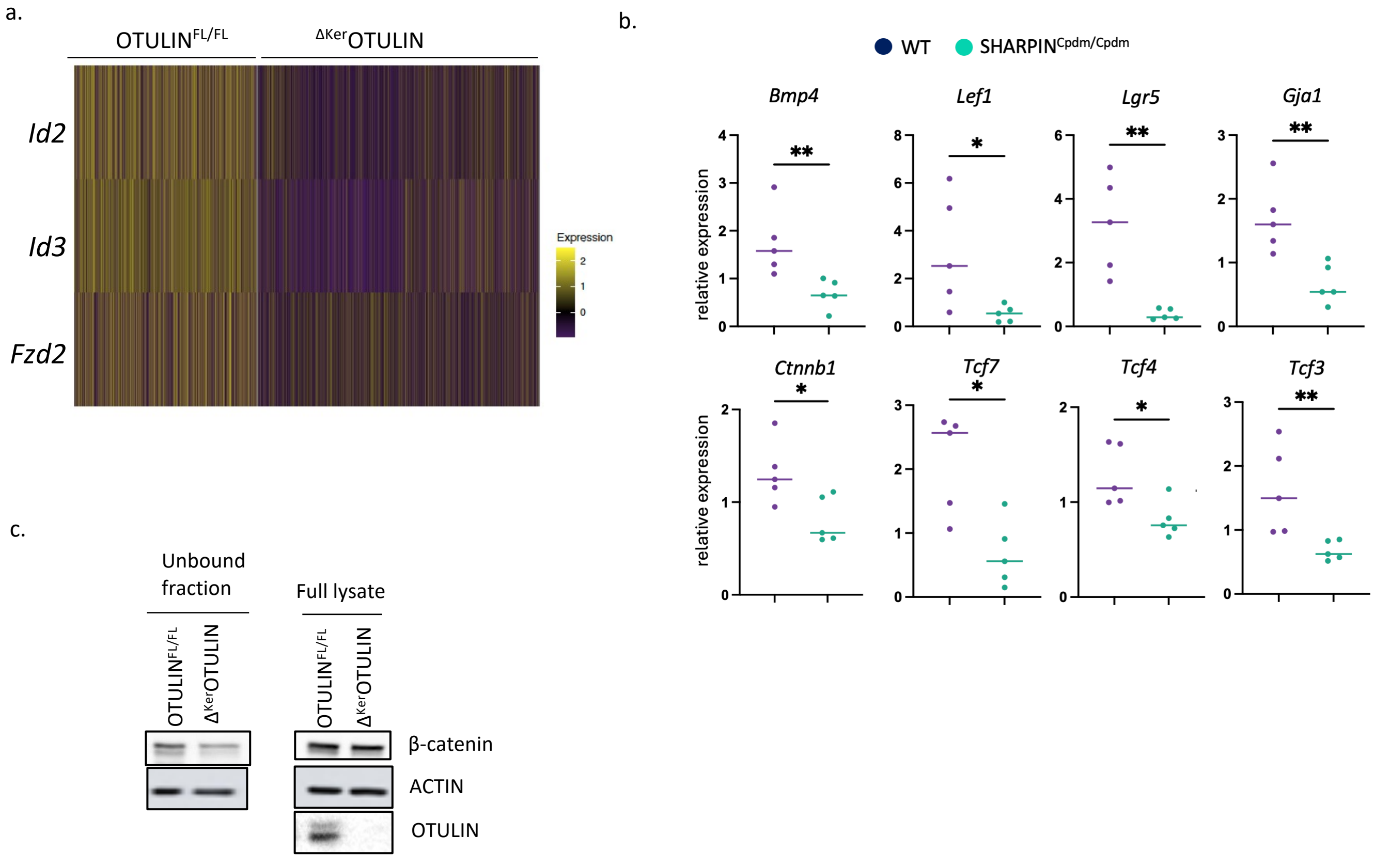

Supplementary Figure 1. Induction of canonical Wnt signalling in KCs. a. Heatmap showing the expression of the indicated genes in scRNAseq data from KCs isolated from OTULIN$^{FL/FL}$ and Δ^{Ker}OTULIN skin. Each line represents a single KC. **b.** Quantitative PCR analysis for the indicated Wnt mediators and target genes on epidermal tail lysates of wild-type and SHARPIN$^{cpdm/cpdm}$ mice (aged 8-11 weeks). Data are means ±SEM, $^{*}p< 0.05$; $^{**}p< 0.01$. **c.** Western blot analysis of the ConA-unbound fraction and full epidermal tail lysates from OTULIN$^{FL/FL}$ and Δ^{Ker}OTULIN mice. α-actin blot is shown as loading control.

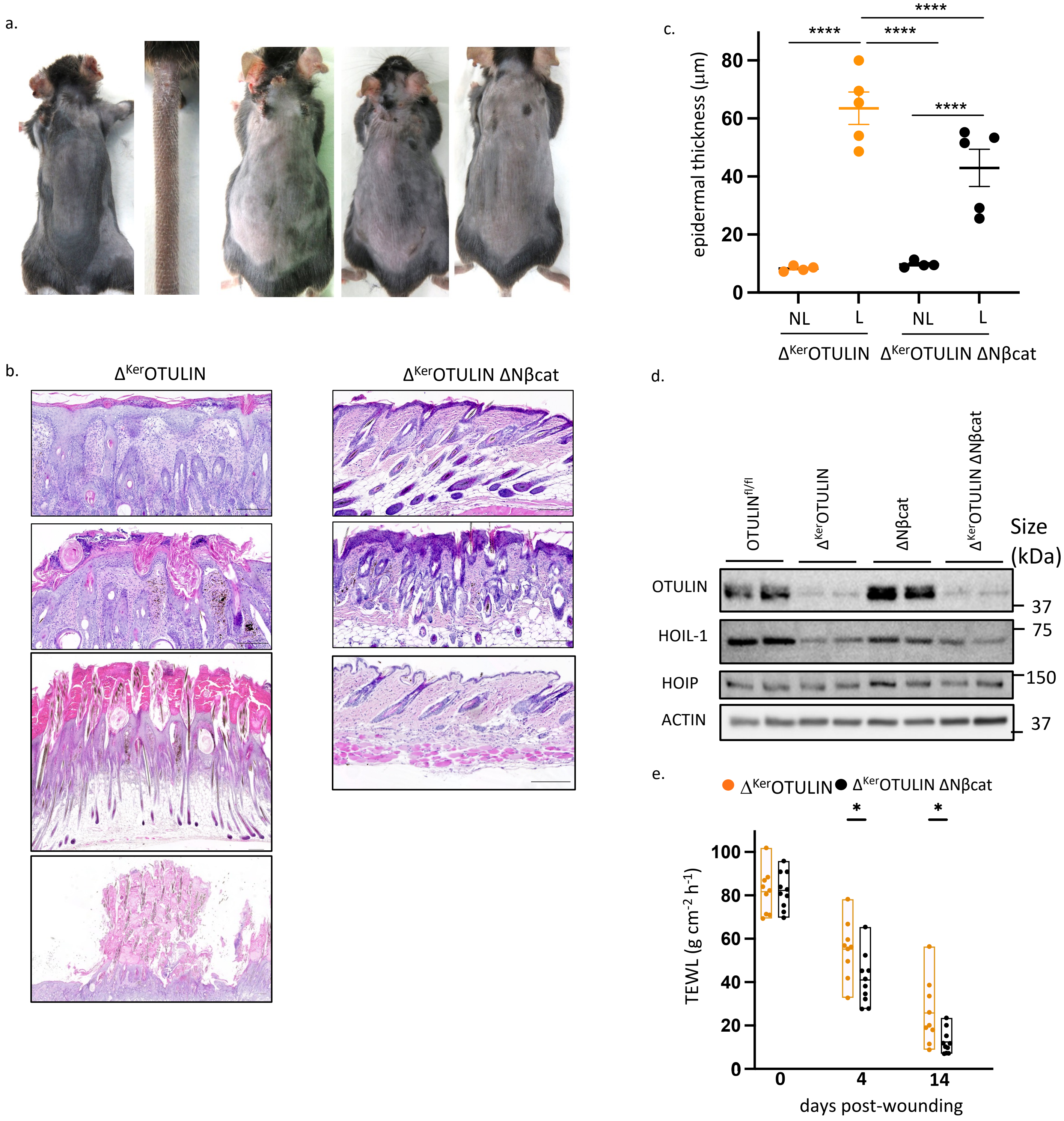

Supplementary Figure 2. Constitutive activation of canonical Wnt signalling protects Δ^{Ker}OTULIN mice from dermatitis. a. Representative pictures of mouse back and tail skin of Δ^{Ker}OTULIN ΔNβ-cat mice at the age of 40 weeks. **b.** H&E-stained skin sections of 11 weeks old Δ^{Ker}OTULIN and Δ^{Ker}OTULIN ΔNβ-cat mice. Scale bars: 200 μm. **c.** Epidermal thickness measured on H&E-stained skin sections (NL: non-lesional; L: lesional) from tamoxifen-treated Δ^{Ker}OTULIN and Δ^{Ker}OTULIN ΔNβ-cat 11 weeks old mice (n≥ 4 mice per condition, for each biological replicate the mean of 10 measurements was taken; ****$p < 0.0001$; two-way ANOVA with multiple comparisons). **d.** Immunoblotting for OTULIN, HOIL-1 and HOIP in epidermal tail lysates from OTULIN$^{FL/FL}$, ΔNβ-cat, Δ^{Ker}OTULIN and Δ^{Ker}OTULIN ΔNβ-cat mice. α-actin is shown as loading control. **e.** Wound healing kinetics assessed by TEWL measurements after full-thickness wounding in Δ^{Ker}OTULIN (n= 9) and Δ^{Ker}OTULIN ΔNβcat (n= 10) mice (*$p< 0.05$; Two-way ANOVA with multiple comparisons).

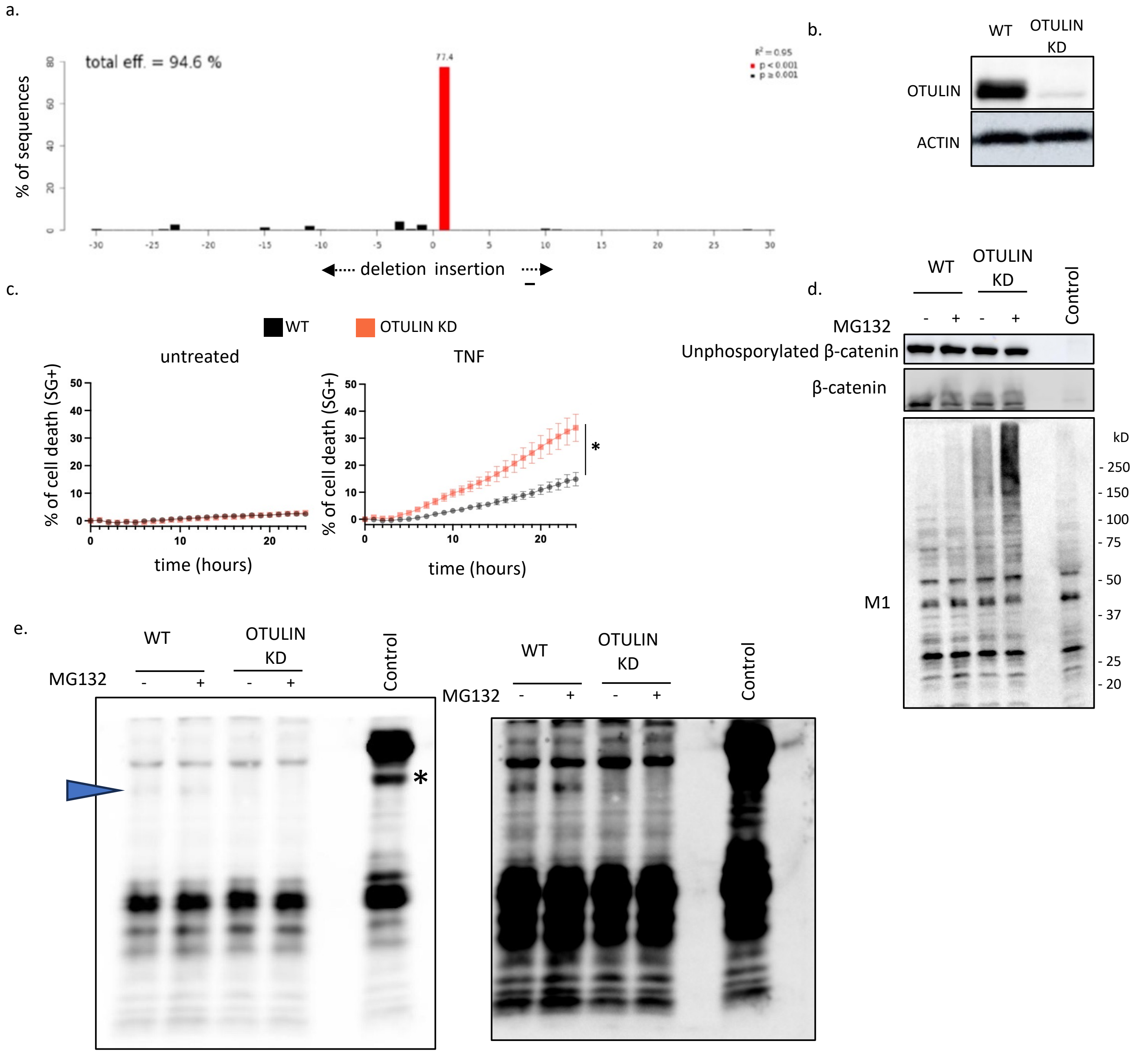

Supplementary Figure 3. Generation of OTULIN-deficient human KCs and immunoprecipitation studies in mouse and human KCs. a. Quantitative spectrum of indels created by two consecutive treatments with OTULIN-targetting nanoblades as assessed by TIDE (Tracking of Indels by DEcomposition) analysis. **b.** Immunoblot for OTULIN on lysates from wild-type (WT) and OTULIN knock-down (OTULIN KD) HaCaTs. α-actin is shown as loading control. **c.** WT and OTULIN KD HaCaTs (n=3 technical replicates per condition) were left untreated or treated with 30 ng/ml hTNF for 24 hrs. Viability was assessed by Sytox Green uptake. Data represent means ± SEM (*p<0.05; REML analysis). **d**. Blots of the immunoprecipitation experiments shown in Figure 4d, including visualization of control (beads only, no IP antibody). **e.** Full blots for OTULIN of the immunoprecipitation experiments shown in Figure 4d, including visualisation of control (beads only, no IP antibody). Asterisk depicts an aspecific band; arrowhead indicates OTULIN-specific band. Short exposure is shown on left, longer exposure on the right.

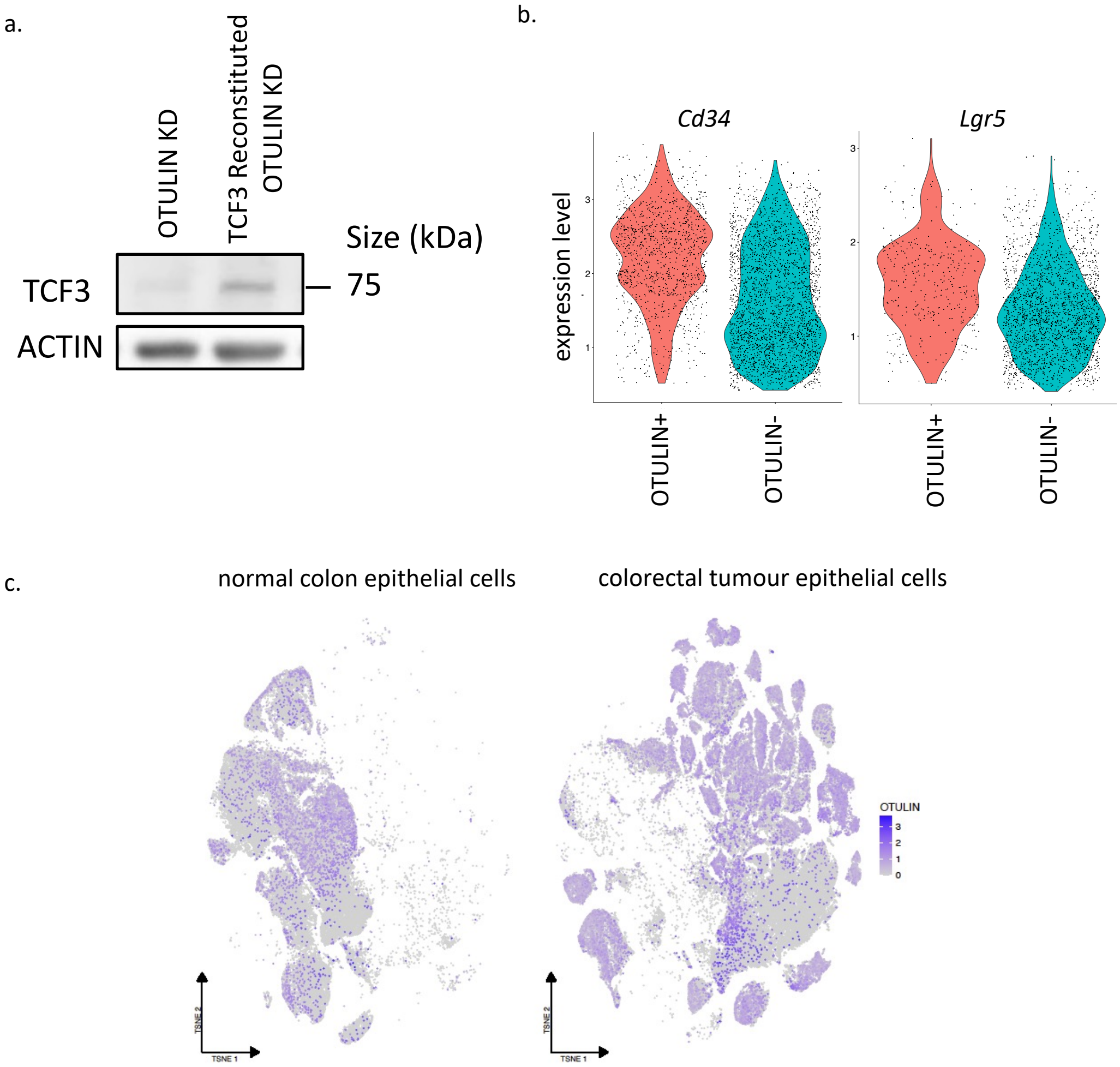

Supplementary Figure 4. OTULIN regulates TCF3/4 levels in health and disease. a. Immunoblotting for TCF3 in OTULIN KD HaCaTs and OTULIN KD HaCaTs reconstituted with TCF3 by lentiviral transduction. α-actin is shown as loading control. **b.** Violin plots showing the normalised expression levels of hair follicle stem cell markers *Cd34* and *Lgr5* in OTULIN-positive versus OTULIN-negative KCs. **c.** Feature plots showing expression levels of *OTULIN* in epithelial cells from normal human colon (left) and in tumour epithelial cells from colorectal cancer (right) as assessed by scRNAseq (36).
